# **Burn injury in Mottahari Burn Center in Tehran, Iran**

**Published:** 2016-01

**Authors:** Fatemeh Mohadeth Ardebili, Mehri Bozorg Nejad, Zahra Sadat Manzari

**Affiliations:** 1 School of Nursing and Midwifery, Iran University of Medical Sciences, Tehran, Iran;; 2 Medical-Surgical Group, Nursing and Midwifery School, Mashhad University of Medical Sciences, Mashad, Iran

**DEAR EDITOR**

Burn is one of the devastating conditions in emergency medicine resulting into physical and psychological disabilities.^1^ During pregnancy, it has an increasing trend for mortality and morbidity of both mother and infant.^2^ So practical activities to decrease its physical and emotional complications seem mandatory.^2^ For survivors, the most persisting problem is scarring while healing is a complex process including inflammation, granulation, and remodeling of the tissue.^3^

Silver sulfadiazine was reported as the gold standard in topical burn therapy with antibacterial properties.^4^ There may be the resistance of several bacteria to silver sulfadiazine. So, there is a need for new agents for treatment of burn wounds with less adverse effects and more efficacy.^4^ The medicinal herbals were extensively used in wound healing of burn injuries.^5^^-^^9^

Elderly patients are more at risk of burn, due to physiological limitations caused by aging, and the immune system problems.^10^ Most of these patients usually have a history of diseases such as cardiovascular, pulmonary, renal and that is because they are subjecting at high risk of burn side effect like deformity, septic shock and reducing the healing time too.^10^

The enrolled population were of both elderly and non-elderly patients, of both male and female genders referring to Motahari Burn center in Tehran, Iran. The questionnaires was completed by burn specialist. The reliability was confirmed by Cronbach’s alpha of 84.0 and statistically analyzed. 

As Table 1 shows, the 60-70 and 12-30 years old patients were the most susceptible age groups and were mostly male. The majority of elderlies lived in Tehran and in non-elderly from other cities. Most of patients in elderly group were illiterate and married. They were mostly unemployed and suffered from poor economic situation. Most of elderly patients had no history of diabetes, hypertension, cardiovascular diseases, hyperlipidemia, asthma, some degrees of visual problem and hearing losses. While in the non-elderly group, most of patients did not have any history of those diseases except for hypertension, diabetes and mental illnesses. 

**Table 1:** Demographic data of burn patients

**Variable**		**Non-elderly**		**Elderly**	
		**Percent**	**Number**	**Percent**	**Number**
Sex	Male	46.3	829	61	82
	Female	53.7	715	38	51
Age (years)	30-12	42.3	654	-	-
	40-31	28.1	435	-	-
	50-41	17.9	277	-	-
	60-51	11.5	178	-	-
	70-60	-	-	9.66	89
	80-71	-	-	27.8	47
	90-81	-	-	0.45	6
	Above 90	-	-	0.07	1
Place of living	Tehran	38.2	590	59.2	79
	City	47.8	739	38.2	51
	Village	13.9	215	2.4	3
Marital status	Single	4	66	10.5	14
	Married	45.6	70	46.7	62
	Divorced	10.6	16	3.8	5
	Widow	0	1	39	52
Education	Illiterate	2.9	45	56.4	75
	Elementary	50.9	787	25.6	34
	Diploma	40.3	623	15.7	21
	Higher	5.7	89	2.3	3
Occupation	Retired	1.1	18	19.6	26
	Employed	17.1	263	6	8
	Private	32.1	496	24.7	33
	Housewife	49.7	767	49.7	66
Economical status	Good	4	62	5.2	7
	Average	38.9	601	30	40
	Poor	57.1	881	64.7	86
Type of living	Owner	53.8	831	41.3	55
	Elderly house	0	0	5.2	7
	With children	2.6	41	16.5	22
	Hired	43.6	672	36.8	49
Living situation	Alone	0.4	6	33.9	45
	With husband	45.6	704	45.1	60
	With child	11.2	173	16.5	22
	Else	42.8	661	4.5	6
Support	Have	97.4	1503	72.9	97
	Not having	2.6	41	27.1	46

As Table 1 demonstrates, most burn time in elderly patients were in the evening and in non-elderly patients in the morning and noon time. The burn season in both elderly patients and non-elderly patients was in the winter and then in the fall for both groups. For most of burn patients, the waiting time for help was about an hour. In the elderly patients, hot liquids and gas were the causative agents for burn injuries, while in non-elderlies group was gasoline and hot liquids. An accident was the major cause of burn inflict and then self-suicides. The degree of burns in elderly patients was grade 2 and 3 while in non-elderly patients was semi-deep or deep. The majority of burns in the elderly patients was in the trunk while in the non-elderly group was in the arms. 

The majority of burn patients in both elderly and non-elderly group were married, unemployed and were in poor economic class and with low educational level. Our findings are in consistent with Cutillas et al. (1998) in southwestern France on 716 patients. They showed more prevalence in males and mostly at homes. Most burns occurred in urban or suburban areas and the mortality was higher among patients living in urban areas.^11^ In another study in the Netherlands on 94 patients aged over 60 years, it was shown that female burn patients were more and mostly at home. Most burns occurred in the limbs and trunk. The average hospitalization time was 1.34±1.30 days and 59% of the patients had a history of previous disease.^10^ Bortolani and Barisoni (2007) showed that of 386 burn cases, 53 patients were older than 60 years old. Most injuries were because of hot liquids, and then gas explosion.^12^

Considering more infliction of burn injuries in elderly, honoring the elderly, improving their quality of life, improving the economic, social and psychological condition of the elderly should have a priority for governmental officials. There is a much more need for taking care of elderly burn patients that may be susceptible to mortality based on their previous diseases.
